# Characteristics and simulation of snow interception by the canopy of primary spruce‐fir Korean pine forests in the Xiaoxing'an Mountains of China

**DOI:** 10.1002/ece3.5152

**Published:** 2019-04-21

**Authors:** Yang Xiao, Xiaosong Li, Shuping Zhao, Guohua Song

**Affiliations:** ^1^ College of Agriculture Resources and Environment Heilongjiang University Harbin China; ^2^ Key Laboratory of Digital Earth Sciences Institute of Remote Sensing and Digital Earth Chinese Academy of Sciences Chinese Academy of Sciences Beijing China; ^3^ Northeast Forestry University Harbin China; ^4^ Heilongjiang Fenglin National Nature Reserve Authority Yichun China

**Keywords:** canopy structure, hydrological models, snow interception, snowfall

## Abstract

Snow interception by the forest canopy is an important control on the forest hydrological cycle in the Xiaoxing'an Mountains within the northern temperate region of China. In this study, the effects of snowfall characteristics and stand structures on the snowfall redistribution of the canopies within primary spruce‐fir Korean pine forests are analyzed at the forest stand scale. Characteristics of snowfall, through‐canopy snowfall, and stand structure are continuously measured using positioning observations. A semiempirical theoretical model is used to conduct snow interception simulations in the Xiaoxing'an Mountain region. The results indicate that the snowfall, canopy density, slope gradient, and tree height have a significant effect on the through‐canopy snowfall. The interception efficiency gradually decreases with an increase in the amount of snowfall and is particularly sensitive to the snowfall and canopy density, although it shows no significant correlation with average diameter at breast height, tree height, basal area, canopy height, canopy width, leaf area, or slope gradient. Very similar results have been observed in Canada and Switzerland, suggesting the transferability of the results between North America, Western Europe, and China. However, although model results provide a satisfactory simulation of snow interception, further studies are required to optimize the model in this region.

## 1 INTRODUCTION

The interception of snow by the forest canopy has important effects on the hydrologic cycle in regions with seasonal snow coverage (Moeser, Morsdorf, & Jonas, 2015; Varhola, Coops, Weiler, & Moore, 2010; Yoichi et al., 2017; Zhang, Zhou, & Cai, 1994). Research on the effects of forest on the processes of snowfall interception has important scientific significance for climate change, forest management, forest fires, and vegetation succession in areas of the forest that experience seasonal snow cover. In highly forested areas, canopy snow interception is the primary control on the amount of snow available for spring melt. In the Xiaoxing'an Mountain of northern China, communities rely on the snowpack as a vital water resource, as up to 30% of annual streamflow is provided by snowmelt alone (Zhang et al., 1994). During periods of snow accumulation, it has been found that as much as 40% of total snowfall can be intercepted by the canopy in the Xiaoxing'an Mountain region (Liu, Cai, Man, Chai, & Lang, 2012; Liu, Cai, Yan, & Bai, 2010; Zhang, Xiao, Zhang, Song, & Wang, 2015). Once the snow is intercepted, it lost by sublimation, evaporation, or drip melt. Snowfall interception processes may represent a potential water loss in terms of snowmelt runoff from forested areas. This is particularly important in the Xiaoxing'an Mountain region where 64.7% of land is forested (Zhang et al., 1994). However, in order to fully understand and predict the effects of forest, we must first be able to accurately investigate the relationship between the mixed spruce‐fir Korean pine forests and the snow interception in the Xiaoxing'an Mountains of China.

The process of snow interception is influenced and restricted by snowfall characteristics, canopy structure, tree species, and the microclimate (Lundberg & Halldin, 1994). Many researchers have conducted extensive studies on this process. In this respect, Miller hypothesized (1964) that snow interception is subject to the morphology characteristic of canopy, air temperature, and wind speed. Tennyson, Ffolliott, and Thorud (1974) used time‐lapse photography to assess potential interception and found that the rate of snowfall interception storage on the uneven‐aged stand of ponderosa pine increases in a nonlinear manner, with initial deposition being rapid, then slowing with time. Fitzharris (1975) suggested that snow interception by forest stands could be described using a linear equation of snowfall, and Strobel (1978) showed that under different stand densities, snow interception efficiency decreases with increasing snowfall. Harestad and Bunnell (1981) observed that canopy density and snowfall have significant effects on the efficiency of snow interception in coastal forests, and although McNay, Petersen, and Nyberg (1988) and Pomeroy and Gray (1995) found no significant differences in interception efficiency under different snowfall conditions, it was notably influenced only by the canopy density. Furthermore, Calder (1990) studied the gamma ray attenuation of fir forests and found that snow interception efficiency is related to the speed and duration of snowfall and that snow interception can be described using a linear equation of snowfall depth. Pfister and Schneebeli (1999) found that air temperature has a significant influence on snow interception efficiency. Marsh (1999) and Garvelmann, Pohl, and Weiler (2013) also suggested that interception efficiency increases with increasing temperature and decreasing wind speed. Storck and Lettenmaier (2002) reported small differences in the maximum capacity of snow interception for different conifer species. Lundberg and Koivusalo (2003) showed that interception loss from gross precipitation increases with increasing forest density and approaches 30% for forests of the highest density class, and the results of Liu et al. (2010), Liu et al. (2012) showed significant differences with respect to snow interception from different types of forests, different snowfall intensities, and snowfall classes. It has also been determined that variations in the characteristics of forests and climate cause greater variations in the snow interception efficiency. Examples of this include the canopies of coniferous forests, which can store 60% of the snowfall in the cold temperate continental climate of North America (Strasser, Warscher, & Liston, 2011), and intercept 32%–35% of the snowfall in the temperate maritime climate of Scotland (Lundberg, Calder, & Harding, 1998), and intercept 12%–40% of the snowfall in the Xiaoxing'an Mountains within the northern temperate continental climate zone of China and in the Daxing'an Mountains (which are within the cold temperate continental climate zone) (Li, Cai, Sheng, & Yu, 2014; Zhang et al., 2015).

The process of snow interception by the forest canopy is relatively complex, but many researchers have established, tested, and optimized canopy interception models. For temperate climate conditions, Satterlund and Haupt (1967) felled and weighed young Douglas firs and white pine trees to observe the process of snow interception and develop an empirical statistical model on a single tree scale. It has been determined that interception efficiency is relatively low for branches of young trees under mild and high snow loads but is higher under moderate snow loads. It is also evident that snow interception is closely related to snowfall and the maximum intercepted snow load by the forest canopy, and in this respect, Schmidt and Glunns (1991) used a method involving cutting of spruce, fir, and black pine branches to weigh and observe the processes of snow interception. They also verified and revised the model of Satterlund and Haupt (1967), and establishing an empirical statistical model for a single branch scale.

The abovementioned models were developed for a single tree and single branch scale, respectively, and were established under temperate maritime climate conditions, where the snow interception preload for individual snowfall events was fixed to 0. However, the process is different under cold temperate continental climate conditions, and snow intercepted by the canopy can remain for days (Pomeroy & Schmidt, 1993); therefore, the snow interception preload is not 0. Hedstrom and Pomeroy (1998) and Pomeroy, Gray, Hedstrom, and Janowicz (2002) felled and weighed trees to develop a semiempirical theoretical model of snow interception at a stand scale based on the physical mechanisms in a cold temperate continental climate. Their model considers the effects on snow interception with respect to the snow stored on the canopy, canopy structure, and snowfall, which have clear physical significances. Andreadis, Storck, and Lettenmaier (2009) established a theoretical model of snow interception based on coupled energy and water balance at a stand scale, which divided the snow intercepted by the canopy into solid states and liquid states. However, to operate this model, multiple meteorological parameters are required for the canopy layer, and the calculations involved are relatively complex.

In summary, three types of models currently exist for canopy interception: (a) empirical models at single branch and single tree scales; (b) semiempirical theoretical models at a stand scale; and (c) theoretical models based on the energy and water balance mechanisms at a stand scale (Xiao, Zhang, & Song, 2017), and all have certain advantages and limitations. In this respect, empirical statistical models are easy to use but contain fewer ecological parameters, which mean that the effects of certain forest canopy characteristics and other factors are ignored with respect to the interception process. Theoretical models require a large number of meteorological parameters, which limits their usage in regions that lack meteorological data; they also involve complex calculations. In contrast, semiempirical theoretical models have a simple structure, require fewer parameters, and can better reveal the mechanisms of canopy interception process; however, although these models can be operated using canopy structure and snowfall parameters, further research is required on the applicability of these models in different climatic zones and types of forests.

The differences between snowfall and rainfall interception mechanisms are relatively large. For example, snow can remain on the canopy for a longer period of time than rain, which is only retained for a short period of time. In China, many researchers have studied the effects on rain interception within classical climate forests relating to nearly every forest types (Chen, Zhang, Yu, Shi, & Huang, 2013; Chen et al., 2015; Li et al., 2013a,2013b; Liu, Sun, & Wen, 2003; Lu et al., 2015; Sun, Wang, Li, Liu, & Lin, 2011), but fewer studies have examined the role that mixed spruce‐fir (*Picea koraiensis* and *Abies nephrolepis*) and Korean pine (*Pinus koraiensis*) forests in northeastern China have on snow interception. However, such studies are important to understand such hydrologic effects within the forest ecosystem in China, where snowfall and snowmelt are the major hydrologic processes.

Korean pine forests are classical and zonal climatic vegetations within coniferous forests in the northern temperate regions and are widely distributed in the Xiaoxing'an and Changbai Mountains of China. Although several researchers have already conducted studies on the snowfall redistribution with respect to Korean pine forests in the Xiaoxing'an Mountains. Liu et al. (2010), Liu et al. (2012) showed that snow interception between different types of *Pin. koraiensis* virgin forest was related to the snowfall, the larger the snow was, the more significant the differences. The interception amount of different forest types was related to snow intensity, and interception rate was also various in different snowfall intensities for the same forest type. Such studies have mainly focused on the relationship between the interception process and the amount and intensity of snowfall, whereas there has been less focus on the effects from the structural characteristics of the forest canopy on the snowfall redistribution.

This study therefore examines the effects of the forest canopy on the snow interception in the mixed spruce‐fir Korean pine forests of the Xiaoxing'an Mountains, with the aim of analyzing the relationship between the structural characteristics of the canopy and dynamic changes in both snow interception and through‐canopy snowfall. The semiempirical statistical model established by Hedstrom and Pomeroy is used to conduct a snow interception analysis, optimize model parameters, and accurately estimate the amount of snow intercepted by forest canopies in this region. The results provide a theoretical basis for the quantitative evaluation of snow‐related eco‐hydrological effects over classical zonal forests in this region.

## 2 MATERIALS AND METHODS

### 2.1 Site description

This study was conducted at the Heilongjiang Xiaoxing'an Mountains Forest Ecosystem Research Station, which is a 18,165‐ha forest reserve located in the middle of the Xiaoxing'an Mountains within the Fenglin reserve of the Heilongjiang province, approximately 60 km north of Yichun City (128°59′–129°15′E, 48°02′–48°12′N). It is managed by the Fenglin State Natural Reserve Department of the Heilongjiang and Chinese Forest Ecosystem Research Network Management Center. The elevation in the natural reserve ranges from 285 to 688 m; the mean gradient ranges from 10° to 25°; and annual precipitation ranges from 680 to 750 mm. Precipitation as rain is mainly distributed between July and August, and between November and March most of the precipitation accumulates as snow: snow accounts for as little as 10%, or as much as 20%, of annual precipitation. Precipitation as rain to snow is distributed between October and November, and between March and April the precipitation accumulates as snow and rain. The average annual temperature and humidity at the research site is approximately −0.5°C and 78%, respectively; and average annual wind speeds range from 2.5 to 5 m/s. The vegetation consists primarily of natural uneven‐aged old‐growth broad‐leaved Korean pine forests and natural secondary white birch forests. White birch (*Betula platyphylla*) and Korean pine (*Pin. koraiensis*) are the dominant tree species in the region, and other species include spruce‐fir (*Pic. koraiensis* and *A. nephrolepis*), birch (*Betula costata*), basswood (*Tilia amurensis*), oak (*Quercus mongolica*), and larix (*Larix gmelinii*).

### 2.2 Experimental design

Experiments were conducted under natural snowfall conditions from November 2013 to April 2015. At the research station, two 1‐hm^2^ plots (with a distance between them of 500 m) were designated for the long‐term and fixed‐point monitoring of snowfall, in which the coniferous trees species were Korean pines, spruce, and fir. The tree height (*H*), diameter at breast height (DBH), canopy height, canopy width, canopy density (*C*_c_), and effective leaf area index (LAI), as well as the slope gradient, slope aspect, and slope position, were extensively examined (Table 1 lists plot characteristics). Meteorological variables were measured on a horizontal surface approximately 4 km from the study site at the Fenglin Meteorological station.

**Table 1:** Plot characteristics

Site	Plot no.	Age (years)	Stand density (stems/ha)	DBH (cm)	Height (m)	Slope (º)	Canopy height (m)	canopy width (m)	canopy density (%)	leaf area index (%)
Spruce‐fir‐Korean conifers	A	150–300	357	40.8	22.7	9.8	16.5	7.5	45	1.96
Spruce‐fir‐Korean conifers	B	150–300	1,032	42.8	25.9	8.9	19	8.9	65	2.19

The monitoring and examination methods employed in this study are described as follows:

1. Measuring amount of snowfall

**Figure 1:**
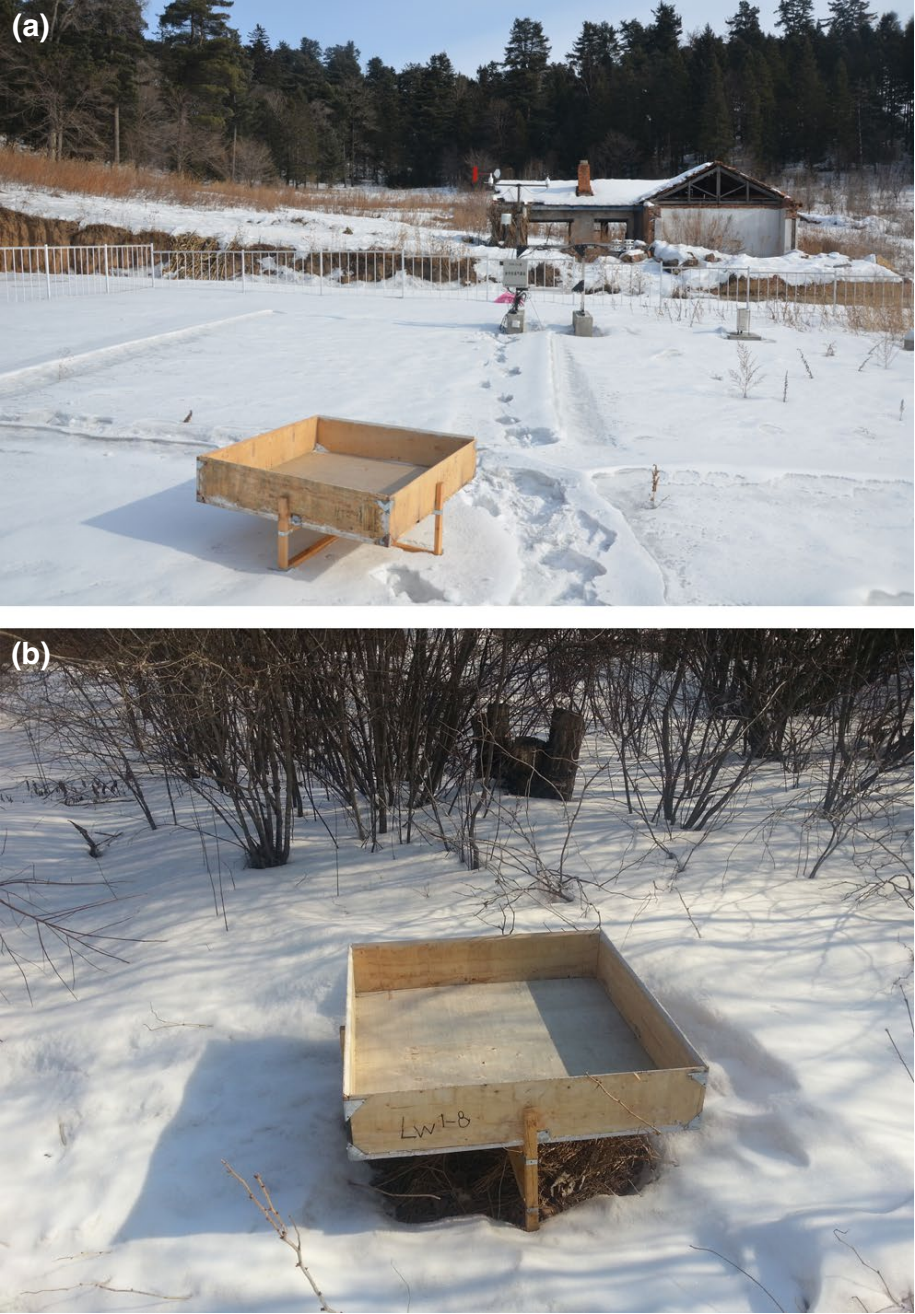
(a, b) Snow troughs in the opening sites

Four snow troughs were at random installed in the opening sites at 20‐m intervals to measure the amount of snowfall. The distance between the opening site and the borders of each plot was 200 m. The area at the bottom of the wooden snow trough is 1 m^2^ with a 20‐cm height at the edges in order to prevent wind from blowing snow off the snow trough. The edges of the snow trough were angled at 45°, so that the snowflakes falling on the edges can slide into the snow trough (Figure 1). The snow troughs were placed on wooden frames at a height of 50 cm above the ground. To decrease the error caused by snow sublimation/evaporation and windblown snow during the measurement process, measurements of snowfall were conducted immediately after each snowfall event. Using a steel ruler, the snow depth was measured at four points at 0.2‐m intervals along the diagonal of the snow trough bottom. Three snow cores were randomly extracted by vertically inserting a 4.6‐cm diameter polyethylene tube (of a known mass) into the snowpack within the snow trough, and snow density was estimated by weighing a known volume of the sampled snow cores. The mean snow water equivalent (SWE) was determined by:

(1) $$\text{SWE}\;\left(\text{mm}\right)=D\times \frac{\rho_{s}}{\rho_{w}}$$

where, *D* is the mean snow depth, $\rho_{s}$ is the mean density of fresh‐fallen snow in the snow trough (g/cm^3^), and $\rho_{w}$ is the density of water (g/cm^3^).

2. Measuring amount of through‐canopy snowfall

**Figure 2:**
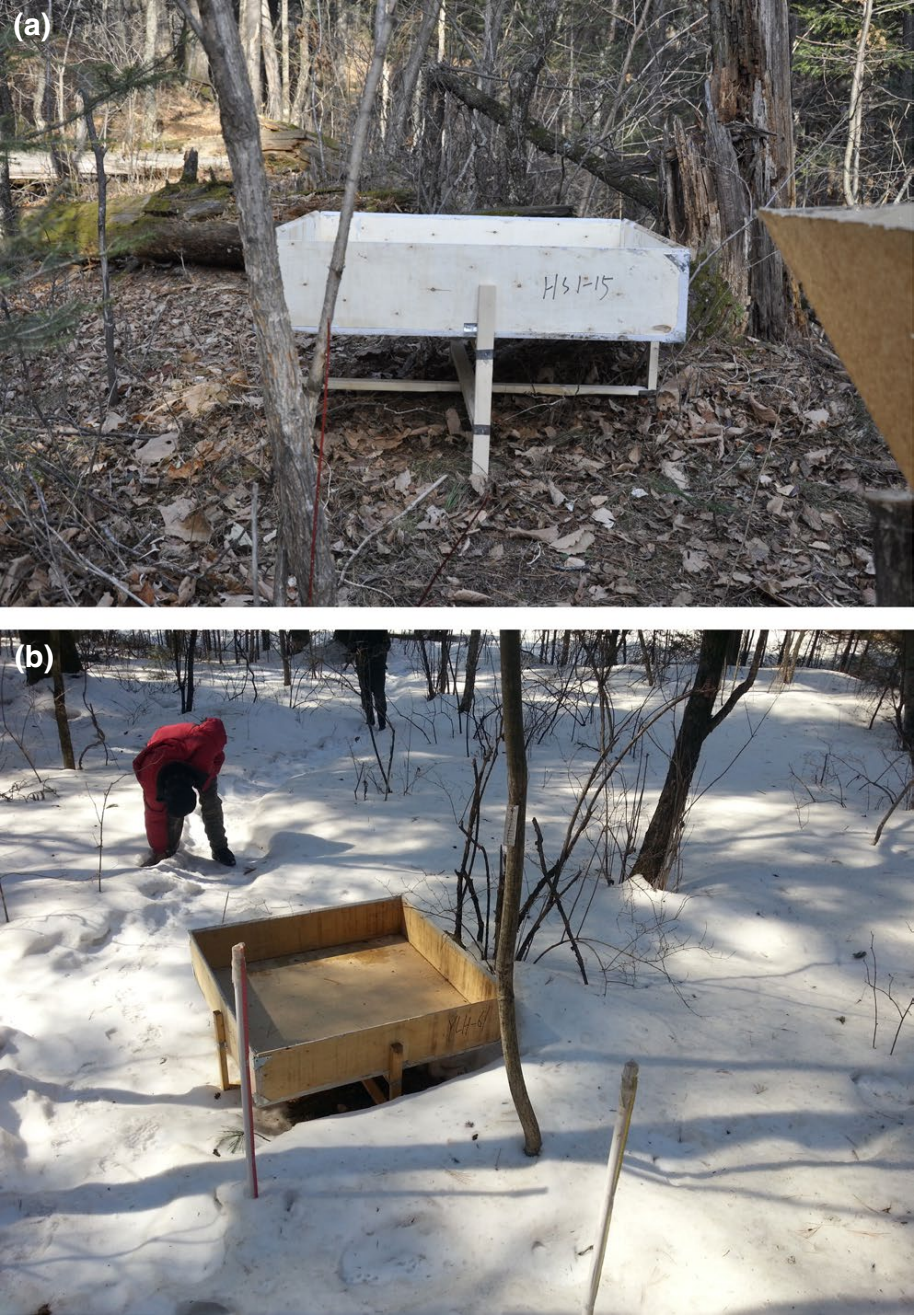
(a, b) Snow troughs in the forested sites

Twenty‐eight snow troughs were set up at 10‐m intervals along the diagonals of the plots (Figure 2). As in the previous case, a steel ruler was used to measure the snow depth and a polyethylene tube was used to collect snow samples to enable calculation of the amount of through‐canopy snowfall in the snow troughs after each snowfall events.

3. Determining amount of the snow intercepted by the canopy

According to the principle of water balance, the amount of the snow intercepted by the canopy (mm), *i*, can be expressed as:

(2) $$i=P_{c}-T$$

where, *P*_c_ is the secondary snowfall amount (mm), and *T* is the amount of through‐canopy snowfall (mm). This equation ignores the amount of evaporation/sublimation of the snow intercepted by the canopy.

4. Obtaining forest metrics, canopy density, and effective LAI

Average tree DBH, basal area, and canopy width were investigated by conducting individual tree measurements conducted in the field during early October 2013. Tree height and canopy height were measured using an ultrasonic wave height indicator (Vertex IV 60), and a leveling instrument was used to measure the slope gradient and slope aspect of plots. The height from the top to the base of the tree and canopy was used as the measurement of tree height and canopy height, respectively. The geometric mean of the minimum and maximum crown diameter was used as the measurement of crown width.

**Figure 3:**
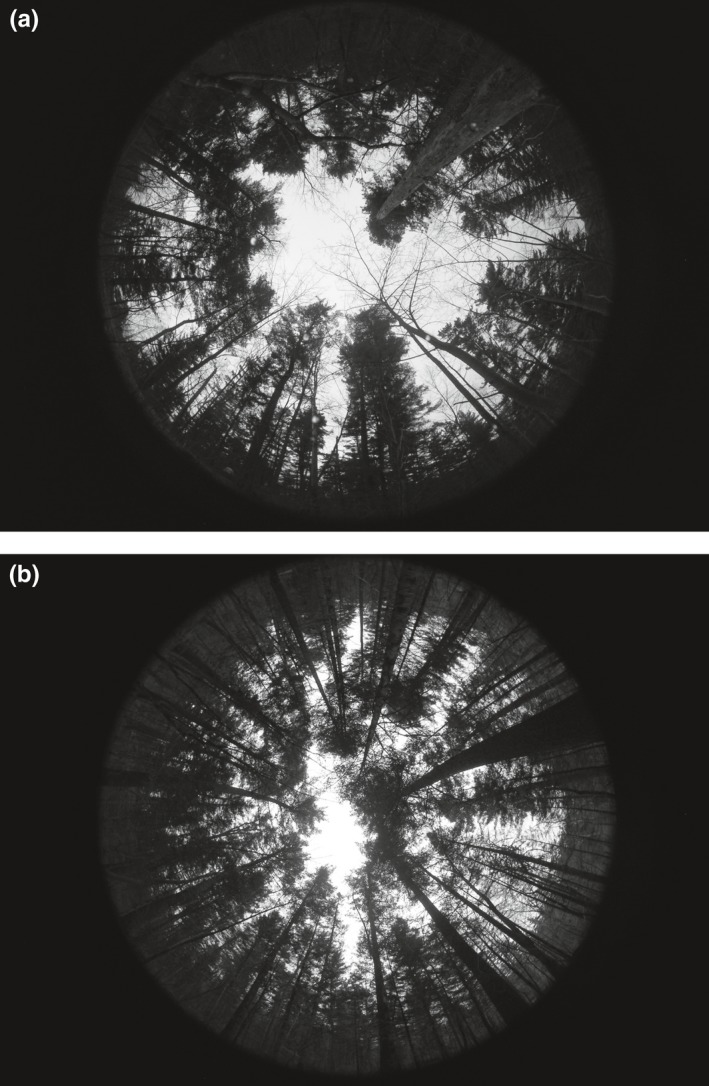
Photographs taken with a hemispherical (fish‐eye) lens at the forested plots (a, left) and plots (b, right)

On cloudy days or after sunset, a Nikon Coolpix 995 (*f* = 7–32 mm) camera, with a Nikon FC‐E8 (*f* = 8–24 m) fish‐eye lens was used to photograph the top of each snow trough within sampling plots (Figure 3). The camera was kept level, and photographs were taken vertically skywards. A minimum focal length was used to capture the maximum photography area through the fish‐eye lens. All‐sky photographs of the stand were taken of a view that excluded those areas outside the sampling plot (Yao et al., 2015), and three hemispherical photographs were taken in each snow trough sampling point. Canopy density and effective LAI was averaged over the three photographs to obtain one value per snow trough sampling point. Fish‐eye images were analyzed by the analysis system of the HemiView canopy to obtain canopy density and effective LAI at the top of each snow trough sampling point.

### 2.3 Interception models

This study used a snow interception model, based on the physical mechanisms applicable to a stand scale, which was established by Hedstrom and Pomeroy (1998) and Pomeroy et al. (2002). This model assumes that the effective LAI, canopy density, and coniferous species can be used to determine the snow interception ability of the canopy. For individual snow interception events, the model takes the following form.

(3) $$i=cI^{*}\left(1-e^{-C_{c}p_{c}/I^{*}}\right)$$

(4) $$I^{*}=S_{\mathrm{pLAI}}\left(0.27+\frac{46}{\rho_{s}}\right)$$

where, *i* is the amount of the snow intercepted by the canopy (mm), *c* is the empirical unloading coefficient of the snow intercepted by the canopy, *p*_c_ is the snowfall amount (mm), *C*_c_ is canopy density, I* is the maximum amount of the snow intercepted by the canopy, *S*_p_ is snow load coefficient, LAI is the effective LAI in winter (total horizontal area of stems, needles, and leaves per unit area of ground), and $\rho_{s}$ is the density of fresh‐fallen snow (g/cm^3^).

### 2.4 Data processing

Statistical analyses were conducted out using SPSS. Simple regressions were used to evaluate trends in through‐canopy snowfall and snow intercepted by the canopy with respect to 17 snowfall events. At each snow trough sampling point, correlation analysis and multiple regressions were computed for the dependence of through‐canopy snowfall and snow intercepted by the canopy on the means of stand variables and terrain factors, which were obtained within a 5‐m radius of the snow trough center (average DBH, average tree height, average basal area, average canopy height, average canopy width, canopy density, effective LAI, and slope gradient were used as independent variables in the analyses).

## 3 RESULTS

### 3.1 Snowfall characteristics during observation period

**Figure 4:**
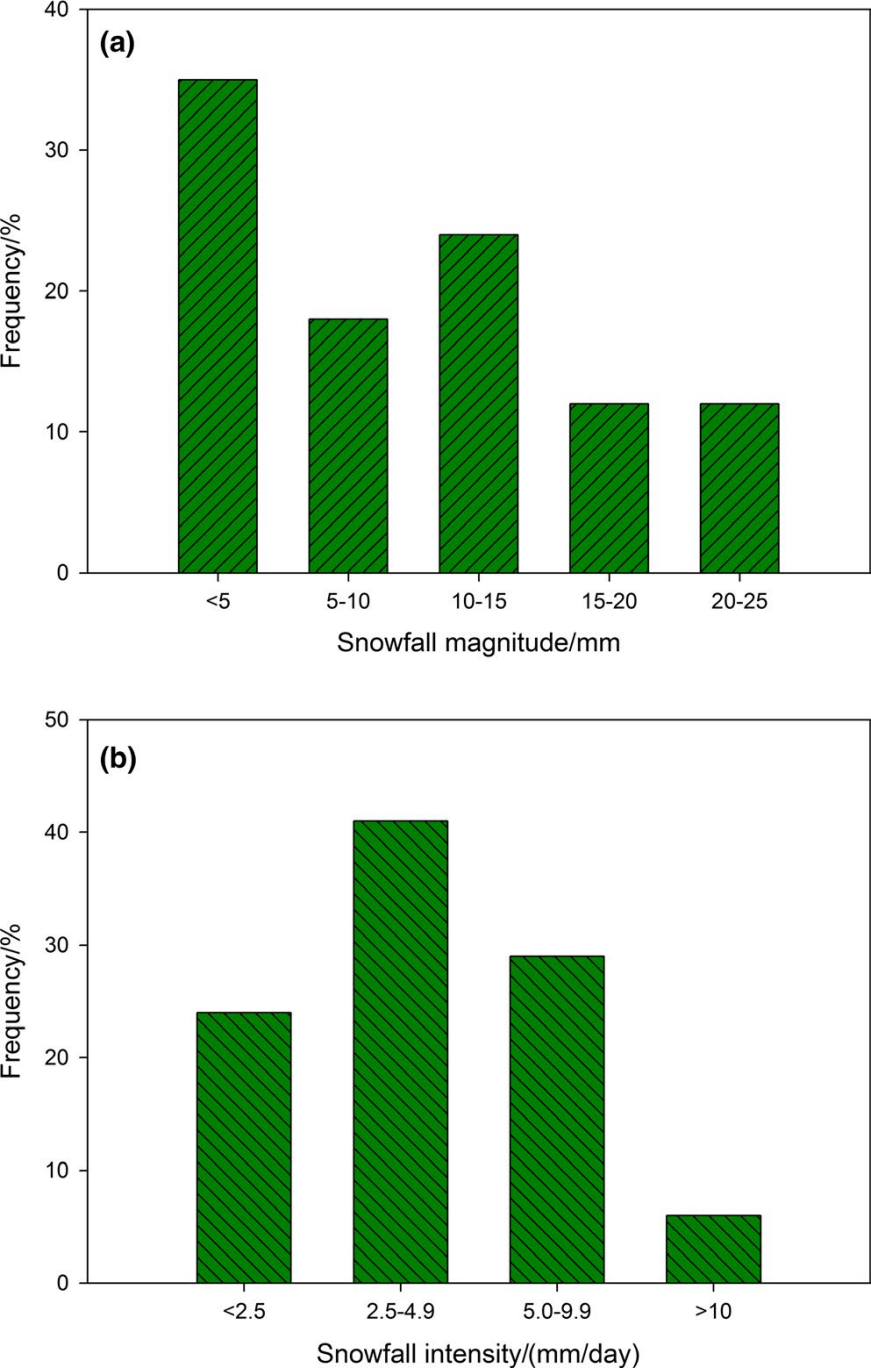
Frequency of snowfall occurrence with (a) different snowfall magnitude and (b) different snowfall intensities during the observed period (2013–2015)

The daily temperature during the storm period ranged from −0.6°C on 13 November 2013 to −30.1°C on 6 April 2015, with a mean of −9.3°C. Conditions from the early of November to end March were very cold, with maximum temperatures only exceeding 0°C by a small amount on a limited number of days. The precipitation accumulated as snowfall only during the storm period. During the period of study from 2013 to 2015, the frequency of snowfall occurrence was 53% for snowfall magnitude lower than 10 mm, but significantly lower for snowfall magnitude greater than 15 and 20 mm (12% in both cases). The average snowfall amount was 10.3 mm, and the variation coefficient of snowfall was 1.01. The mean intensity of snowfall was 4.41 mm/day, with a minimum snowfall intensity of 0.48 mm/day, and a maximum intensity of 10.40 mm/day. The variation coefficient of snowfall intensity was 1.14. For snowfall intensities lower than 4.9 mm/day and greater than 5 mm/day, the frequency of snowfall occurrence was 65% and 35%, respectively (Figure 4). Based on the relevant standards (General Administration of Quality Supervision, Inspection, and Quarantine of the People's Republic of China, & Standardization administration of the People's Republic of China, 2008), the observed results showed that the region experienced moderate and light snowfall.

### 3.2 Characteristics of through‐canopy snowfall and snow intercepted by canopy in spruce‐fir Korean pine forests

#### 3.2.1 Characteristics of through‐canopy snowfall in spruce‐fir Korean pine forests

**Figure 5:**
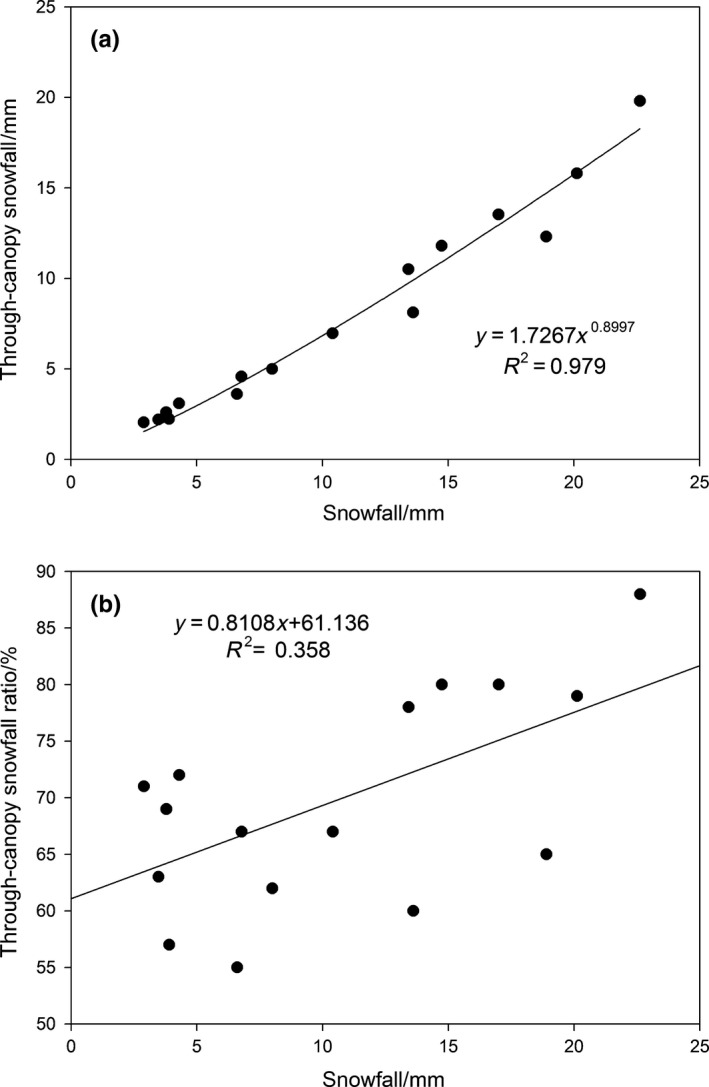
Regression analysis between snowfall and through‐canopy snowfall (a), and the ratio of through‐canopy snowfall (b) during 17 snowfall events from 2013 to 2015

After each snowfall event, the through‐canopy snowfall in 28 snow troughs was investigated during each snow season, and the average amount of through‐canopy snowfall of the 28 snow troughs was used to represent the amount of through‐canopy snowfall of the entire stand. During the study period, a total of 17 snowfall events were observed with a cumulative snowfall amount of 174.3 mm and cumulative amount of through‐canopy snowfall of 126.2 mm (72.4% of total snowfall amount). The maximum amount of through‐canopy snowfall was 19.8 mm, which represents 85.5% of the maximum amount of snowfall; and the minimum amount of through‐canopy snowfall was 2.0 mm, which represents 70.6% of the snowfall amount. The through‐canopy snowfall percentage is defined as a ratio of the amount of through‐canopy snowfall in the forest to the snowfall amount in the opening for individual snowfall events. The average amount and the ratio of through‐canopy snowfall for 17 snowfall events measured were 7.4 mm and 69.3%, respectively. The amount and ratio of through‐canopy snowfall also increased in accordance with an increase in the snowfall grade. Simple regressions were used to evaluate the relationship between the amount of through‐canopy snowfall and the ratio of through‐canopy snowfall and snowfall form 17 snowfall events, and regression analysis showed evidence of a significant power function correlation between the amount of through‐canopy snowfall and snowfall (*R* ^2^ = 0.979), with the ratio of through‐canopy snowfall and snowfall showing a positive correlation (*R* ^2^ = 0.358; Figure 5).

#### 3.2.2 Characteristics of snow interception in spruce‐fir Korean pine forests

**Figure 6:**
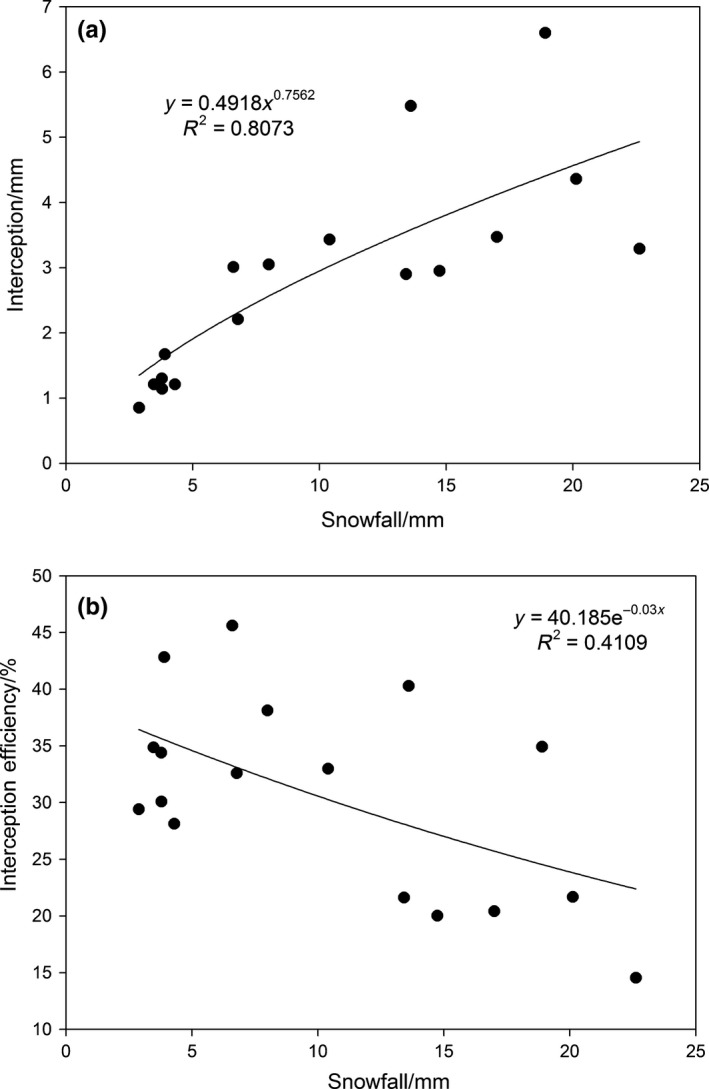
Regression analysis between snowfall amount and (a) the amount of snow interception, and (b) the efficiency of snow interception during 17 snowfall events from 2013 to 2015

The average amount of snow intercepted by the canopy was calculated according to Equation (2) using the amount of through‐canopy snowfall and snowfall measured in 28 snow troughs after each snowfall event. The average amount of the snow intercepted by the canopy represents the snow interception of the stand as a whole. Figure 6 shows that during the observed period, the total amount of the intercepted snow by the canopy was 48.1 mm, representing 27.6% of the total snowfall amount. The maximum amount of snow interception at a single snowfall event was 6.6 mm, with an interception efficiency of 34.9%, where interception efficiency is the ratio of the amount of snow interception to snowfall in an individual snowfall event. The minimum value observed was 0.85 mm with an interception efficiency of 29.4%. The average amount of the snow intercepted by the canopy was 2.8 mm, and the average interception efficiency was 30.7%. Simple regressions were used to evaluate the relationship between snow interception, snow interception efficiency, and snowfall in 17 snowfall events. Snow interception and snowfall showed a strong power function correlation (*R* ^2^ = 0.8073), but snow interception efficiency and snowfall showed a negative exponent function correlation (*R* ^2^ = 0.4109).

### 3.3 Correlation analysis between through‐canopy snowfall and snow intercepted by the canopy and stand structure characteristics and terrain factors at 56 snow trough sampling points in spruce‐fir Korean pine forests

After each snowfall event, the amount of through‐canopy snowfall in each snow trough was investigated and calculated. Stand variables and terrain factors were investigated precisely within a 5‐m radius of each snow trough center in early October 2013 (Table 2) at each snow trough sampling point. The average amount of through‐canopy snowfall in each snow trough was also calculated after seven snowfall events from 13 November 2013 to 4 February 2014 and ten snowfall events from 3 November 2014 to 6 April 2015; this amount represents the amount of through‐canopy snowfall of trees within a 5‐m radius of each snow trough center. A correlation analysis was conducted between through‐canopy snowfall and snow intercepted by the canopy and stand structure characteristics and terrain factors at 56 snow trough sampling points in the spruce‐fir Korean pine forests (from 2013 to 2015).

**Table 2:** Average amount of through‐canopy snowfall and snow intercepted by the canopy with forest characteristics and slope recorded at 56 snow trough sampling points in spruce‐fir Korean pine forests (from 2013 to 2015)

Snow trough numbers	Average through‐canopy snowfall (mm)	Average interception (mm)	Numbers of coniferous stem	Canopy density (%)	LAI	DBH (cm)	BA (cm)	Canopy height (cm)	Canopy width (cm)	H (cm)	Slope (%)
1	9.6	2.1	2	47.6	2.41	43.2	63.4	21.4	8.6	26.3	0
2	9.5	2.2	2	54.6	2.13	59.0	109.3	27.5	12.0	33.5	0
3	9.4	2.3	4	43.9	1.50	48.3	23.0	22.5	10.2	28.4	0
4	8.1	3.6	6	60.5	2.08	43.3	74.2	18.5	7.2	22.9	29
5	9.5	2.3	4	53.1	2.05	32.1	45.1	13.8	6.4	20.4	22
6	8.2	3.5	5	63.6	2.90	36.7	53.0	21.9	8.2	27.6	17
7	8.2	3.5	3	63.8	2.50	40.8	64.0	19.1	10.2	24.5	8
8	10.8	1.1	2	45.5	1.42	67.7	144.3	18.6	9.6	26.6	25
9	10.1	1.7	1	31.0	1.09	62.4	122.3	18.9	11.3	27.4	21
10	10.4	1.4	3	39.2	1.46	55.0	100.2	22.9	9.9	30.1	5
11	9.4	2.4	2	51.2	2.56	60.0	114.5	20.9	11.4	28.4	17
12	9.7	2.0	6	56.2	2.60	31.4	53.9	12.3	6.1	20.5	38
13	9.5	2.2	2	55.1	2.77	46.9	79.2	14.5	7.8	20.8	38
14	9.7	2.0	2	36.3	3.44	34.5	53.4	8.9	5.5	13.0	56
15	10.3	1.4	4	62.4	3.03	54.2	92.8	16.9	10.3	25.8	56
16	9.5	2.2	3	25.8	0.73	8.7	2.9	3.5	2.9	6.2	56
17	8.5	3.2	3	45.3	1.40	44.5	68.3	17.6	8.5	24.7	56
18	7.1	2.0	4	54.2	1.65	38.3	54.2	14.4	9.5	23.6	31
19	6.9	2.6	3	52.8	2.16	47.3	40.0	20.0	8.0	26.0	31
20	6.9	2.5	4	69.3	2.58	38.4	55.6	15.6	8.8	25.3	31
21	7.4	3.0	3	56.3	2.27	29.5	45.3	12.3	7.6	18.7	31
22	8.2	2.3	5	53.2	2.04	42.3	62.0	15.1	10.3	23.0	49
23	7.7	3.0	2	42.4	1.28	59.2	113.2	14.5	9.1	21.9	49
24	7.9	4.3	5	45.3	1.33	32.0	32.2	16.3	8.3	22.8	49
25	7.4	4.7	1	53.0	1.67	59.8	112.3	20.7	11.4	30.0	49
26	7.8	3.0	2	57.1	4.33	51.4	128.5	17.1	9.5	20.6	35
27	7.3	3.3	1	44.2	1.48	29.7	27.7	20.8	7.6	24.0	35
28	6.2	3.1	3	64.5	3.67	51.1	84.4	16.9	10.2	24.0	35
29	5.8	2.8	8	68.0	3.22	20.3	17.9	9.6	5.4	12.4	35
30	6.2	3.2	2	52.4	2.19	43.2	63.4	21.4	8.6	26.3	0
31	6.5	2.7	3	57.0	2.74	37.5	36.0	17.5	9.0	26.9	0
32	6.1	3.1	7	62.6	3.01	27.0	44.2	9.9	6.1	16.5	0
33	6.5	2.8	3	49.9	2.26	28.5	31.8	13.6	5.9	19.5	0
34	5.9	3.3	3	46.1	1.50	54.1	105.1	17.0	10.6	27.0	0
35	6.2	3.1	3	58.5	2.16	35.5	67.2	14.3	8.9	23.4	0
36	4.7	4.6	4	63.7	2.64	36.8	102.5	21.9	11.0	29.8	0
37	7.5	1.7	1	23.7	0.63	49.0	75.4	16.0	9.2	26.4	35
38	6.3	2.9	1	47.4	1.81	41.0	61.8	17.9	8.6	25.3	34
39	5.8	3.5	2	42.6	1.47	32.3	34.0	12.9	9.0	17.7	30
40	5.7	3.6	4	58.6	2.22	35.3	51.0	14.8	8.6	20.5	23
41	5.3	4.0	5	63.3	2.32	41.6	65.0	19.8	8.5	25.7	23
42	5.2	4.1	6	65.5	3.14	39.4	60.8	19.3	8.7	24.5	10
43	5.5	3.8	3	60.9	2.78	40.8	64.0	19.1	10.2	24.5	8
44	5.5	3.8	8	72.0	2.77	42.0	73.3	15.7	23.6	8.1	0
45	5.9	3.4	4	65.5	4.11	37.1	56.0	15.5	22.2	7.4	7
46	5.9	3.3	5	55.6	1.99	35.2	56.8	14.4	20.2	6.9	17
47	7.7	1.5	1	40.5	1.50	60.0	90.0	23.0	10.0	29.0	0
48	6.1	3.1	3	51.9	1.75	34.5	52.6	14.5	18.0	7.1	21
49	7.0	2.2	2	38.2	1.33	41.6	54.9	17.9	23.9	8.2	26
50	7.8	1.4	2	46.3	1.85	18.0	10.5	11.1	14.9	5.9	37
51	6.0	3.3	3	54.7	2.04	43.9	69.1	22.7	29.1	9.9	0
52	7.7	1.6	1	30.6	1.40	57.6	104.2	26.7	37.2	10.0	0
53	6.7	2.5	4	49.5	2.78	38.1	52.4	13.7	22.2	6.1	37
54	7.0	2.3	2	51.2	2.56	60.0	114.5	20.9	28.4	11.4	17
55	6.8	2.4	2	41.7	1.91	69.4	154.4	23.3	35.6	9.3	17
56	6.2	3.0	4	65.2	3.36	47.6	85.2	14.8	25.8	8.5	9

The correlation analysis showed a positive correlation between through‐canopy snowfall and DBH, canopy density, H, and slope gradient, but a negative correlation with BA, average canopy height, average canopy width, and LAI (Table 3). The highest correlation coefficient of −0.44 was achieved between through‐canopy snowfall and canopy density, and the lowest correlation coefficient of 0.264 between through‐canopy snowfall and the DBH. Snow interception was positively correlated with the canopy density, with the highest correlation coefficient of 0.51. However, snow interception was not significantly correlated with DBH, BA, H, average canopy height, average canopy width, LAI, or slope gradient.

**Table 3:** Correlation analysis between through‐canopy snowfall and snow intercepted by canopy, and forest characteristics and terrain factors in spruce‐fir Korean pine forests

Forest type	Forest metric and terrain factor	Through‐canopy snowfall	Interception
Spruce‐fir Korean pine forest	DBH	0.26 (*)	−0.24 (*n*.s.)
	Canopy density	−0.44 (**)	0.51 (**)
	H	0.27 (*)	0.04 (*n*.s.)
	BA	0.19 (*n*.s.)	−0.14 (*n*.s.)
	Canopy height	0.05 (*n*.s.)	0.02 (*n*.s.)
	Canopy width	−0.25 (*n*.s.)	−0.07 (*n*.s.)
	LAI	−0.24 (*n*.s.)	0.24 (*n*.s.)
	Slope	0.32 (*)	0.46 (*n*.s.)

Note

The symbol of ** denotes a significance level of 1%; the symbol of * denotes a significance level of 5%, and “*n*.s.” implies “no significant correlation.”

### 3.4 Regression analysis of through‐canopy snowfall and snow intercepted by canopy, and stand structure characteristics and terrain factors at 56 snow trough sampling points in spruce‐fir Korean pine forests

Through‐canopy snowfall is influenced by snowfall and is closely associated with stand characteristics. Multiple regression analysis showed that through‐canopy snowfall was positively correlated with canopy density, H, and slope gradient (Table 4). The amount of through‐canopy snowfall increased with a decrease in canopy density and an increase in the H and slope gradient.

**Table 4:** Stepwise multiple regression results for through‐canopy snowfall as dependent variable

Sample stratification	b_0_	*C*_c_ b_1_	*H* b_2_	*S* b_3_	*R* ^2^	*S_Y X_*	Sig.	*n*
All data	10.72	−0.063	—	—	0.439	1.43	0.00	56
	9.62	−0.065	0.058	—	0.527	1.36	0.02	56
	8.6	−0.057	0.063	0.023	0.587	1.31	0.03	56

Note

*C*_c_: canopy density; *H*: tree height; *S*: slope gradient; Sig: significance index; *S_Y X_*: standard error of regression.

The multiple regression equation for interception is represented by

(5) $$i=0.728+0.039C_{c}\;\left(R^{2}=0.513,\;S_{Y\;X}=0.73,\;p<0.001\right)$$

and stepwise multiple regression analysis indicates that snow interception and canopy density also have a significant positive correlation. Furthermore, canopy density is found to be the single most significant forest variable explaining snow interception by forest canopy.

### 3.5 Snow interception simulation

#### 3.5.1 Model parameters

Snow interception of the stand as a whole was used to construct a model of snow interception during 17 snowfall events from 2013 to 2015. In the model, the original value set by Pomeroy and Hedstrom (*c* = 0.68) was used as the empirical unloading coefficient of snow interception. Schmidt and Gluns found that the snow load coefficient, S_p_, of pines and spruces to be 6.6 and 5.9 kg/m^2^, respectively, and Pomeroy and Hedstrom used an average value of the snow load coefficient for pines and spruces when constructing their model (*S*_p_ = 6.3 kg/m^2^). Korean pines were the dominant tree species in the sample plots in this study and represented the highest canopy level, while spruces and firs were auxiliary species representing the lower canopy level. Therefore, the snow load coefficient, *S*_p_, was also set at 6.3 kg/m^2^. For snowfall density, Pomeroy and Hedstrom applied the empirical value of 70 kg/m^3^ to the model, which was based on the relationship between snowfall density and air temperature as constructed by the U.S. Army Corps of Engineers. In this study, snowfall density at the study site used actual measured values of  = 81 kg/m^3^. After calibrating of the model parameters, the original model of $i=3.94\mathrm{LAI}\left(1-e^{-C_{c}p_{c}/5.8\mathrm{LAI'}}\right)$ was changed to $i=3.59\text{LAI}\left(1-e^{-C_{c}p_{c}/5.28\text{LAI'}}\right)$ and used in simulations (see Table 5).

**Table 5:** Interception models for the original and the revised cases

Model	Coefficient of determination	Simulation number
$i=3.94\text{LAI}\left(1-e^{-C_{c}p_{c}/5.8\text{LAI}}\right)$	0.800	17
$i=3.59\text{LAI}\left(1-e^{-C_{c}p_{c}/5.28\text{LAI}}\right)$	0.803	17

**Figure 7:**
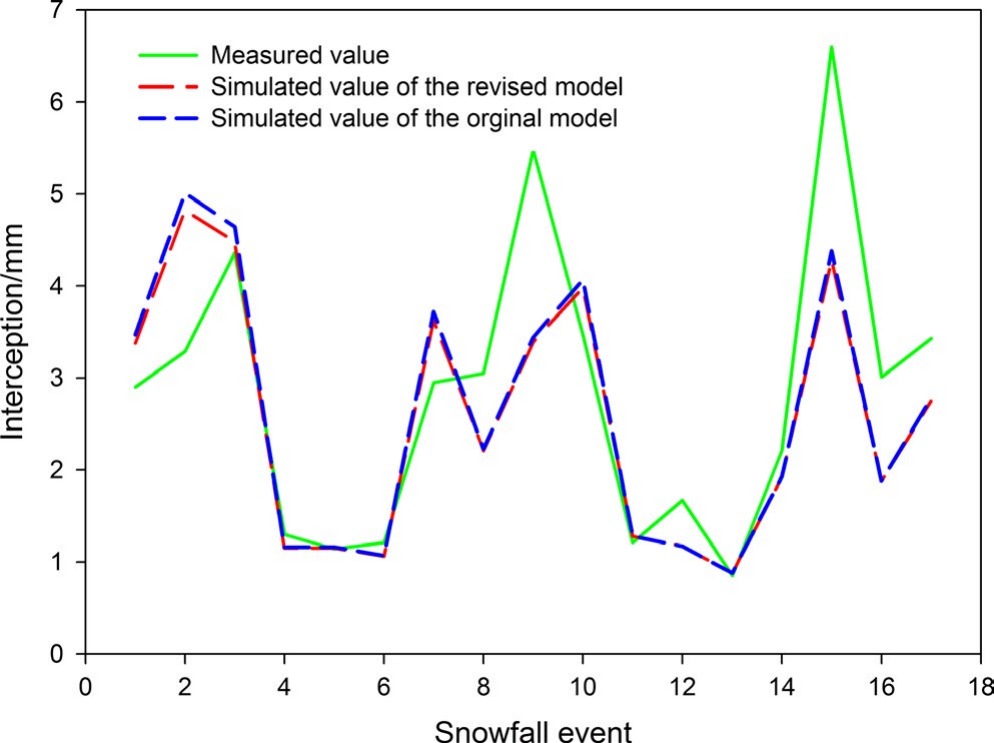
Model simulation effect of canopy interception

According to simulation results using the Pomeroy and Hedstrom model, snow interception from 17 snowfall events was 44.3 mm and the actual amount of snow interception was 48.1 mm. Therefore, the simulated value was lower than the actual value by 3.8 mm. However, the amount of snow interception in the revised model was 43.4 mm, which is 4.7 mm lower than the actual value. The determination coefficient of the regression, *R* ^2^, in the model by Pomeroy and Hedstrom and the revised model were 0.796 and 0.803, respectively; these results are consistent and relatively accurate (Table 5 and Figure 7). From the original model and the revised model, when the amount of snow interception was lower than 2 mm, the simulated and observed values showed good correspondence; however, when the amount of snow interception exceeded 2 mm, the differences between the values were larger.

## 4 DISCUSSION

### 4.1 Relationship between through‐canopy snowfall and snowfall and forest metrics in spruce‐fir Korean pine forests

The amount of through‐canopy snowfall increased with an increase in the snowfall grade, and through‐canopy snowfall and snowfall showed a significant positive power function correlation. There was a simple linear positive correlation between the ratio of through‐canopy snowfall and snowfall. The determination coefficient of the regression, *R* ^2^, in the simple linear regression model was relatively low at 0.358 (Figure 5b). Snowfall size explained only 35.8% of the variation in the rate of through‐canopy snowfall, with the factors such as vegetation characteristics and wind also influencing the process of through‐canopy snowfall.

Results of correlation analysis and stepwise multiple regression in this study show that through‐canopy snowfall is adequately related to the canopy density, tree height, and slope gradient (Tables 4 and 5). However, the relatively high standard error of stepwise multiple regressions (Table 5) indicates that forest variables provide an insufficient input toward the predictive model of through‐canopy snowfall. Therefore, the parameters such as snowfall, air temperature, wind speed, and humidity should be added to predictive model of through‐canopy snowfall should be considered in future studies.

### 4.2 Relationship between the interception and the snowfall and forest metrics in spruce‐fir Korean pine forests

**Figure 8:**
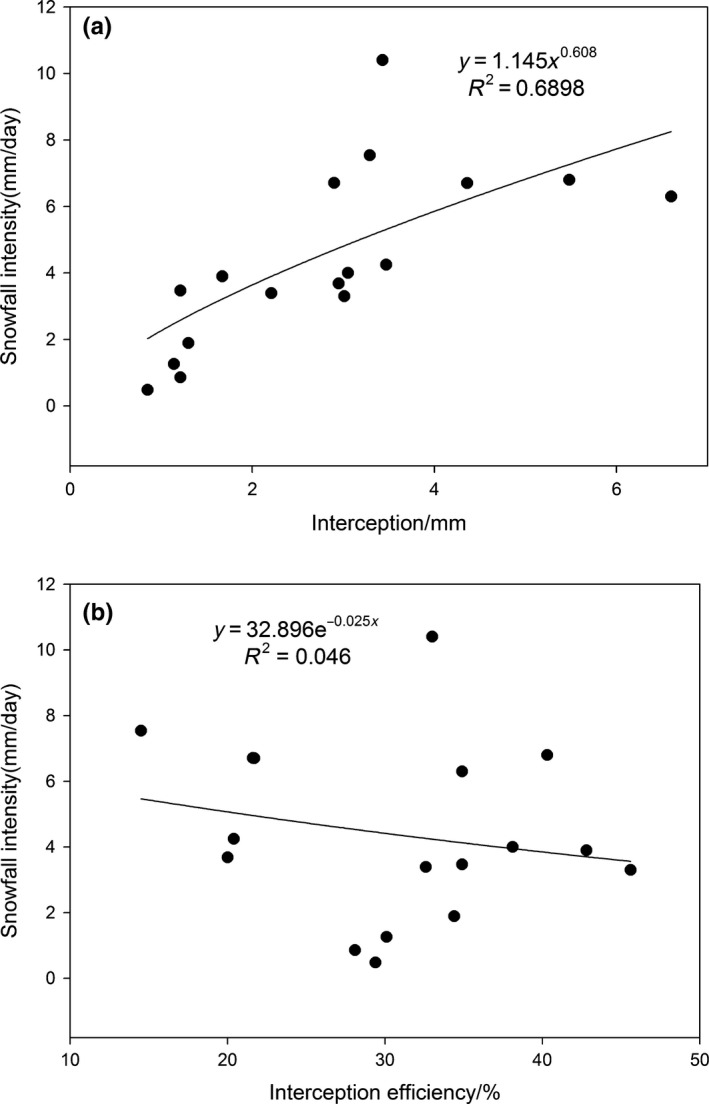
Regression analysis between (a) snowfall intensity and interception and (b) interception efficiency during 17 snowfall events recorded from 2013 to 2015

Interception capacity of forest canopy changed based on snowfall classes, and interception was also strongly associated with snowfall. When the observed snowfall class was lower than 20 mm, the amount of snow interception gradually increased with an increase in snowfall, but when the observed snowfall class was greater than 20 mm, the amount of the intercepted snow by the canopy decreased with an increase in snowfall amount (Figure 6a).When snowfall reached to 18.9 mm on 24 February 2015, the maximum amount of snow interception in 17 snowfall events was 6.6 mm with an interception efficiency of 34.9% (Figures 6and 8a), but when snowfall reached 22.6 mm on 22 November 2013, the amount of snow interception was only 3.3 mm. Stand structure was very similar both these snowfall events, although the amount of snow interception was very different (6.6 and 3.3 mm, respectively). Therefore, the maximum amount of snow was not intercepted when snowfall was more than 18.9 mm.

Under similar stand structure condition, the interception efficiency is largely controlled by wind speed, snowfall density, and temperature (Schmidt & Gluns, 1991). It is evident that the interception process was synthetically influenced by the forest stand characteristics and local climate. Under different temporal and spatial conditions, there was a larger range in the interception efficiency variation between the various snowfall events, and the interception process thus changed dynamically. In this study, a larger difference in the interception efficiency was observed under different snowfall grades. For snowfall grades lower than 10 mm, the average interception efficiency was 35.1%; when the snowfall grade ranged between 10–20 mm, the average interception efficiency slowly decreased to 28.4%; and for snowfall grade larger than 20 mm, the average interception efficiency rapidly decreased to 18.1%. The interception efficiency significantly decreased with an increase in the snowfall class until it finally trended to a stable value (Figure 6b). This demonstrates the limitations of the forest canopy in its capacity to intercept snow.

Snowfall intensity changes constantly during the process of snowfall, and the interception capacity of forest canopy also alters correspondingly. In this study, the observed interception increased with an increase in the snowfall intensity during individual snowfall events (Figure 8a). Regression analysis indicates a significantly positive power correlation between snowfall intensity and interception (*R* ^2^ = 0.6898, *p* < 0.05), but not with interception efficiency (Figure 8b).

This study of snow interception in the spruce‐fir Korean pine forests shows that canopy density has a significant effect on interception efficiency, and this result is consistent with those of Strobel (1978) and Harestad and Bunnell (1981). In this study, the average canopy density of spruce‐fir Korean pine forests was 55% and the average interception efficiency was 31%. However, in a related study by Liu et al. (2012), the average canopy density of spruce‐fir Korean pine forests was 87% and the average snow interception efficiency increased to 39.7%. This study showed a decreasing trend for interception efficiency with an increase in the snowfall amount, where the amount of snow interception was significantly influenced by canopy density. However, the current study results also indicate that the process of snow interception of spruce‐fir Korean pine forests is mainly influenced by the amount of snowfall and canopy density in the Xiaoxing'an Mountains.

### 4.3 Interception model

**Figure 9:**
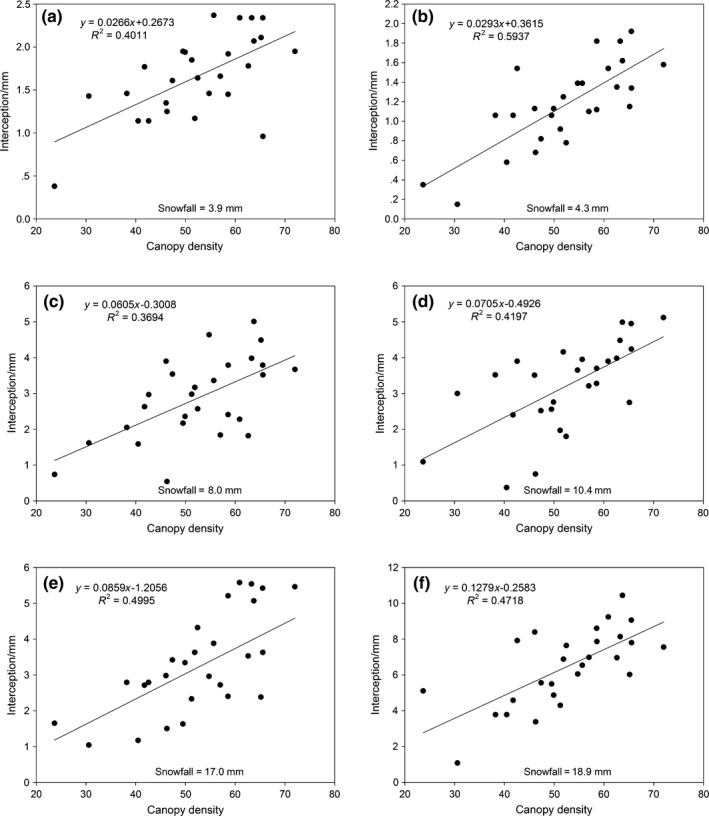
Simple linear regression results of interception as a function of canopy density in different snowfall events

The snow interception model of forest canopy has an important role in understanding of the hydrological process involved in snow interception and redistribution. The characteristics of snow interception by the vegetation canopy are influenced by the amount of snowfall, air temperature, wind speed, humidity, air pressure, and the vegetation characteristics during the snowfall period. However, these parameters also limit the applicability of the canopy interception model to a great extent. In this study, the results of the correlation analysis and stepwise multiple regression showed that canopy density is the single most significant forest canopy variable in the prediction of snow interception. However, the relatively high standard error of stepwise multiple regressions (Equation (5)) indicates that canopy density by itself is an insufficient input for the predictive model of interception. For some snowfall events, the determination coefficients of the regression (*R* ^2^) in the simple linear regression model were relatively low (Figure 9), and therefore, the model of Pomeroy and Hedstrom was used to conduct interception simulations.

The Pomeroy and Hedstrom model is a semiempirical theoretical model which considers for canopy density, effective LAI, and the amount of snowfall. Simulated results based on the cumulative amount of the intercepted snow by the canopy were relatively accurate. The simulated snow interception results for individual events showed that the data are better represented when the amount of snowfall is relatively small, thereby resulting in higher model precision (Figure 7). Therefore, model parameters, such as the snowfall amount and canopy structure, better explain most of the snow interception information, but the remaining information is explained by air temperature, wind speed, humidity, and air pressure. Although the model disregards the sublimation/evaporation effects on the intercepted snow by the canopy during the observation period, the parameters included, such as air temperature, wind speed, humidity, and air pressure, and other microclimatic factors have a significant effect on the sublimation/evaporation process (Knowles, Blanken, Williams, & Chowanski, 2012; Li et al., 2013a,2013b; Lundberg et al., 1998). This may be attributed to the measured interception values of some snowfall events being larger than their simulated values. In addition, the empirical unloading coefficient and the snow load coefficient in the model were obtained from the observations of interception processes in the coniferous forests of North America. Therefore, long‐term monitoring and studies of the coniferous ecosystem in the current region are required to improve the aforementioned coefficient and to optimize the model so that it is region‐specific. This will provide a foundation for the analysis of the dynamic rules of snow interception and related causative mechanisms.

## CONFLICT OF INTEREST

The authors declare no conflict of interest.

## AUTHOR CONTRIBUTIONS

Yang Xiao and Xiaosong Li outlined the research topic, assisted with manuscript writing, and coordinated the revision activities. Shuping Zhao and Guohua Song collected data, performed data analysis, interpreted of results, wrote the manuscript, and coordinated revision activities.

## Data Availability

Source code files of the models: Figshare https://doi.org/10.6084/m9.figshare.6463157
